# Unusual presentation of bilateral pyosalpinx mimicking an ovarian torsion: A case report

**DOI:** 10.1016/j.amsu.2020.02.003

**Published:** 2020-02-26

**Authors:** Sameer Sendy, Allyn Abuy, Wd Sendy, Saeed Baradwan

**Affiliations:** a Department of Obstetrics and Gynecology, Aster Sanad Hospital, Riyadh, Saudi Arabia; b King Abdullah University Hospital, Princess Nourah Bint Abdulrahman University, Riyadh, Saudi Arabia; c HealthPlus Fertility and Women's Health Center, Jeddah, Saudi Arabia

**Keywords:** Pyosalpinx, Tubal abscess, Pelvic inflammatory disease, Ultrasound, Salpingostomy, Case report

## Abstract

**Introduction:**

A pyosalpinx is the acute inflammation of the Fallopian tube fills up and swells with pus, which commonly results from inadequate or delayed treatment of pelvic inflammatory disease. Herein we report a case of bilateral pyosalpinx mimicking an ovarian torsion.

**Presentation of case:**

We reported the case of a 27-year-old female patient, who presented to the emergency department with complaints of constant, worsening lower abdominal pain for 2–3 days. Pelvic and transvaginal ultrasound examinations were performed which demonstrated a large, complex cystic structure in the right adnexa with peripheral flow on color Doppler imaging. The possibilities included ovarian torsion or hemorrhagic cyst. Intraoperative findings showed bilateral pyosalpinx and treated successfully by laparoscopic bilateral salpingostomy.

**Conclusion:**

The present case highlights the diagnostic dilemma of bilateral pyosalpinx must be taken into account in the differential diagnosis of ovarian torsion or tumor, particularly in women of reproductive age.

## Highlights

• Bilateral pyosalpinx is a unique clinical challenge.
• Bilateral pyosalpinx must be taken into account in the differential diagnosis of ovarian torsion endometriosis, dermoid cysts or other cystic ovarian masses.

## 1 Introduction

Pyosalpinx or tubal abscess is an obstruction of the Fallopian tube, resulting in pus accumulation, which commonly results from the spread of bacteria from the lower genital tract. It is a serious complication from untreated or inadequately treated acute pelvic inflammatory disease associated with high morbidity [1].

The diagnosis of pyosalpinx is a unique clinical challenge, Although it's not a rare entity. Transvaginal ultrasound is the initial imaging modality of choices of the diagnosis of a tubal abscess, Although the sonographic appearance of an adnexal mass sometimes overlaps with other entities, including ovarian torsion, endometriosis, dermoid cysts or other cystic ovarian masses [2]. We report a rare case of bilateral pyosalpinx mimicking an ovarian torsion by sonographic imaging. It was successfully treated by laparoscopic bilateral salpingostomy.

## 2 Case description

This Evidence Based Case-Report is made in line with the SCARE criteria [3]. A 27-year-old Asian woman, para 1, presented to our institution with a history of severe lower abdominal pain for 2–3 days. The pain was described as constant, stabbing pain, radiating from the back and thigh, interfering with the patient's daily normal activity, associated with nausea and not relieved by analgesics. There was neither fever nor vaginal discharge. There was no history of vomiting, other gastrointestinal symptoms, urinary symptoms, sexually transmitted disease, and no fainting. The patient had had a low transverse cesarean section (unexplained intrauterine fetal demise) 12 years previously. She had no history of any significant illness or allergies or infertility. There was no significant psychosocial history. Her menarche commenced at the age of 13 years with subsequent regular cycles. She is separated from her husband and denies any recent sexual history.

She was first brought by her employer to another clinic where an abdominal ultrasound showed hemorrhagic right ovarian cysts, probably ruptured, measuring 77 mm × 44 mm. She was asked to transfer to a tertiary hospital. She experienced the same pain 10 days prior associated with on and off undocumented fever. However, this pain is milder in severity. She had the same episodes of pain 1 year ago and was brought to another hospital given unraveled medications. Significant family history of diabetes on her maternal side, no other history of malignancies. She works as a house helper.

On physical examination, she was alert, severe pain, pale-looking, with normal vital signs. An abdominal examination showed right lower abdominal direct tenderness, no distention, and normal bowel sounds. The external genitalia were normal looking. A pelvic examination showed minimal bleeding, normal looking cervix, with wiggling tenderness and bilateral adnexal tenderness. Her complete blood count showed slightly elevated white blood cells (14× 10^9^/L). Hemoglobin, hematocrit, platelet, renal and liver function tests were all within the normal ranges. Urine culture and high vaginal swab results were negative. The serologic chlamydia test was negative. HIV tests were negative. C- reactive protein equal to 15 mg/L and ESR around 30 mm/hour were both elevated.

**Fig. 1:**
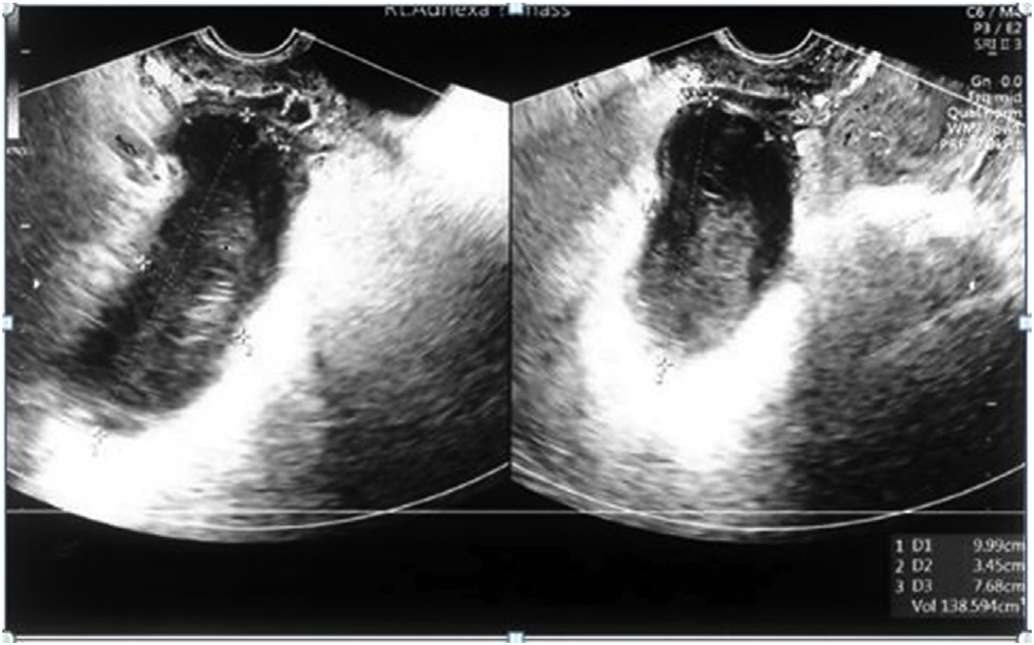
Ultrasound showing an elongated hypo-echoic thick-walled tubule-cystic mass measuring 9.9 x 3.4 × 7.7cm with complex heterogeneous with internal echoes in the right adnexa.

An abdominal and transvaginal ultrasound showed in the right adnexa separate and superior to the right ovary and extending to the mid of the pelvis, there are an elongated hypo-echoic thick wall tubule-cystic masses measuring 9.9 x 3.4 × 7.7 cms with complex heterogeneous with internal echoes. The colour and pulse waves Doppler is positive around the mass. The mass was suggestive of right ovarian torsion. There is a small amount of free fluid measuring 7.0cm³ noticed in the posterior cul de sac [Fig. 1]. Based on these findings along with the patient's symptoms the differential diagnosis was torsion of the right ovarian cyst or hemorrhagic cyst. The patient was counseled and signed informed consent for laparoscopic ovarian cystectomy, possible salpingo-oophorectomy. Intraoperative findings showed distorted pelvic anatomy. There was omental adhesion towards the anterior and left lateral abdominal wall.

**Fig. 2:**
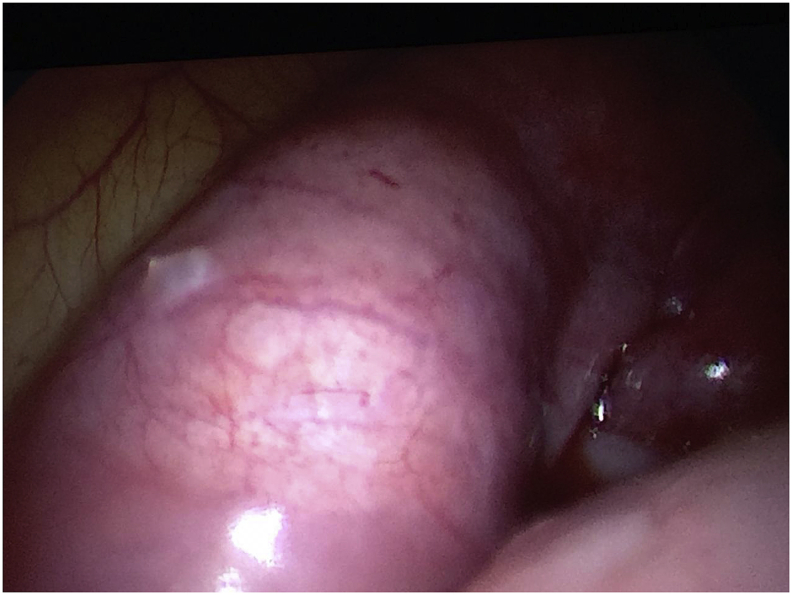
The fallopian tubes were enlarged with a clubbed fimbrial end.

**Fig. 3:**
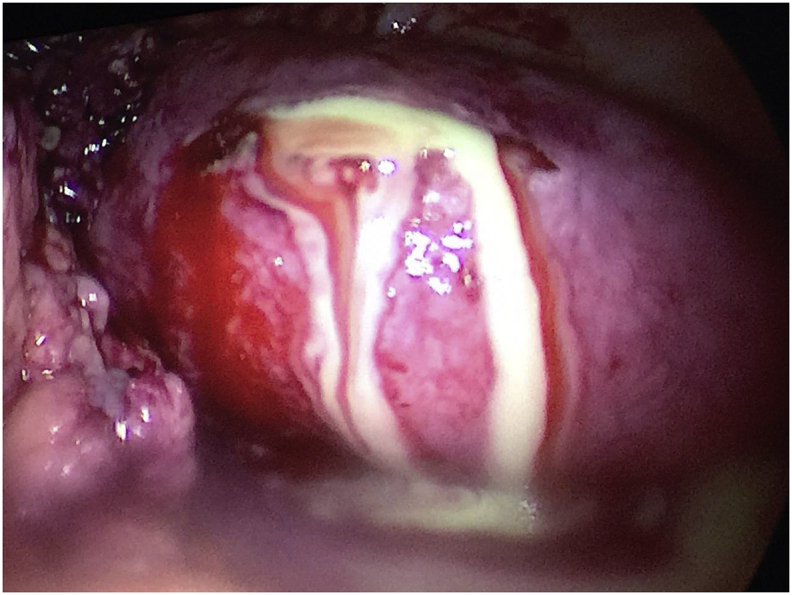
After salpingostomy, the fallopian tubes were filled with pus.

After adhesiolysis, the uterus was seen grossly normal looking together with both ovaries. The left and right Fallopian tubes were enlarged upon a clubbed fimbrial end [Fig. 2]. The right Fallopian tube is bigger than the left Fallopian tube, measuring 10 x 3 × 7 cms. The surgical team decided to proceed with salpingostomy for both tubes. The tubes were filled with pus, samples for culture and sensitivity was taken [Fig. 3]. Diagnosis by this time was made as a case of bilateral Tubal abscess and pelvic inflammatory disease. The pus was drained and peritoneal washing was done. An abdominal drain was inserted. There were no intraoperative complications. The patient was placed on triple antibiotics Ceftriaxone 1 gm IV every 12 hours, Metronidazole 500mg IV every 8 hours and Doxycycline 100mg tablet twice daily. She remained stable postoperative, no fever episodes and her pain subsided. There was no growth after 48 hrs incubation from the culture of the pus.

She was discharged on postoperative day 4 on oral antibiotics for 10 days. The patient went back home with following up in our gynecology clinic. During follow up, her symptoms were improved and performing her normal daily activities. Consent for publication of the report and the image was obtained from the patient.

## 3 Discussion

Pyosalpinx may present with very few specific symptoms or remains silent. The most common presenting symptom is lower abdominal pain, that was present in our patient [2]. Only 50% of women with pyosalpinx present with fever and chills, these symptoms were absent from our patient. Other symptoms include nausea, vaginal discharge, and abnormal vaginal bleeding. On physical examination, patients may show tenderness over the adnexal region with or without guarding or rebound. The absence of specific symptoms and conclusive signs during the physical examination may delay a proper diagnosis.

Predisposing factors in the development of pyosalpinx include sexual activity, multiple sexual partners, nulliparity, previous episodes of pelvic inflammatory disease, lower socioeconomic status and the use of intrauterine devices [4]. It is a serious complication of pelvic inflammatory disease, with high associated morbidity. Though in our case no cultured organisms were identified, the causative infection is usually poly microbial [5]. Bilateral pyosalpinx have been reported to present at menarche in females with underlying urogenital malformations [6] and another case with Hirschsprung's disease [7].

Primary pyosalpinx is one of the most severe complications from pelvic inflammatory disease which characterized by inflammation of the upper genital tract, including endometritis, salpingitis and pelvic peritonitis [8]. Secondary pyosalpinx can rarely arise from infectious or inflammatory processes of the adjacent pelvic structures such as the appendix, colon, and bladder or in association with a pelvic malignancy [9].

The initial imaging modality of choices of the diagnosis of pyosalpinx is transvaginal ultrasound, due to its cost-effectiveness and allows detailed visualization of pelvic structures [10]. Although the sonographic appearance of an adnexal mass can be highly suggestive of a pyosalpinx, there is often an overlap between appearances of another diagnosis, including ovarian torsion, endometriosis, hemorrhagic cysts or other cystic ovarian masses. In our case the finding of a large bilateral pyosalpinx and its confusion with ovarian torsion or tumors. Several imaging is helpful in differentiating between a pyosalpinx and other pathology, including contrast enhanced computed tomography scan and magnetic resonance imaging. In addition to imaging modalities, a thorough history and clinical examination, as well as laboratory studies can play an important role in differentiating these entities. Pyosalpinx is requiring prompt diagnosis, admission, intravenous antibiotics and, possibly aspiration or surgery [11]. Treatment of pyosalpinx varies from conservative management with IV antibiotic to laparoscopic aspiration, image-guided aspiration or drainage, laparoscopic salpingostomy, or salpingectomy [12]. In our case, despite the surgery encountered for the initially presumed ovarian torsion, irrigation and drainage of the abdomen and pelvis after laparoscopic bilateral salpingostomy, in addition to IV and oral antibiotics were sufficient for complete resolution. A randomized clinical trial in 2008 demonstrated that vaginal sonographic drainage with IV antibiotics resolved symptoms quicker than IV antibiotics alone [13].

## 4 Conclusion

We have reported a unique case of bilateral pyosalpinx must be taken into account in the differential diagnosis of ovarian torsion or tumor, particularly in women of reproductive age.

## Ethical approval

Ethical approval has been exempted by our institution for reporting this case.

## Sources of funding

No funding was sought or secured in relation to this case report.

## Author contribution

All authors have contributed to conception and design of the study, drafting the article, revising it critically for important intellectual content.

All authors have approved the final article.

## Research registration number

Not applicable.

## Guarantor

Saeed Baradwan, Sameer Sendy.

## Consent for publication

Written informed consent was obtained from the patient for the publication of this case report.

## Provenance and peer review

Not commissioned, externally peer reviewed.

## Patient consent

Written informed consent was obtained from the patient for the publication of this case report.

## Declaration of competing interest

The authors declare that they have no conflict of interest regarding the publication of this case report.

## Abbreviations

IV: Intravenous
HIV: Human immunodeficiency virus
ESR: Erythrocyte sedimentation rate
MRI: Magnetic resonance imaging
